# Radiomics signature for dynamic changes of tumor-infiltrating CD8+ T cells and macrophages in cervical cancer during chemoradiotherapy

**DOI:** 10.1186/s40644-024-00680-0

**Published:** 2024-04-23

**Authors:** Kang Huang, Xuehan Huang, Chengbing Zeng, Siyan Wang, Yizhou Zhan, Qingxin Cai, Guobo Peng, Zhining Yang, Li Zhou, Jianzhou Chen, Chuangzhen Chen

**Affiliations:** 1 Department of Radiation Oncology, Cancer Hospital of Shantou University Medical College, Shantou, P.R. China; 2 Department of Radiation Oncology, Zhongshan City People’s Hospital, Zhongshan, P.R. China; 3 Shantou University Medical College, Shantou, P.R. China; 4 Department of Gynecologic Oncology, Cancer Hospital of Shantou University Medical College, Shantou, China; 5 Gustave Roussy Cancer Campus, Villejuif Cedex, France; 6 Institut National de la Santé Et de la Recherche Médicale (INSERM) U1015, Équipe Labellisée - Ligue Nationale contre le Cancer, Villejuif, France

**Keywords:** Radiomics signature, CD8+ T cells, Macrophages, Immunomarker classifier, Concurrent chemoradiotherapy

## Abstract

**Background:**

Our previous study suggests that tumor CD8+ T cells and macrophages (defined as CD68+ cells) infiltration underwent dynamic and heterogeneous changes during concurrent chemoradiotherapy (CCRT) in cervical cancer patients, which correlated with their short-term tumor response. This study aims to develop a CT image-based radiomics signature for such dynamic changes.

**Methods:**

Thirty cervical squamous cell carcinoma patients, who were treated with CCRT followed by brachytherapy, were included in this study. Pre-therapeutic CT images were acquired. And tumor biopsies with immunohistochemistry at primary sites were performed at baseline (0 fraction (F)) and immediately after 10F. Radiomics features were extracted from the region of interest (ROI) of CT images using Matlab. The LASSO regression model with ten-fold cross-validation was utilized to select features and construct an immunomarker classifier and a radiomics signature. Their performance was evaluated by the area under the curve (AUC).

**Results:**

The changes of tumor-infiltrating CD8+T cells and macrophages after 10F radiotherapy as compared to those at baseline were used to generate the immunomarker classifier (AUC= 0.842, 95% CI:0.680–1.000). Additionally, a radiomics signature was developed using 4 key radiomics features to predict the immunomarker classifier (AUC=0.875, 95% CI:0.753-0.997). The patients stratified based on this signature exhibited significant differences in treatment response (*p* = 0.004).

**Conclusion:**

The radiomics signature could be used as a potential predictor for the CCRT-induced dynamic alterations of CD8+ T cells and macrophages, which may provide a less invasive approach to appraise tumor immune status during CCRT in cervical cancer compared to tissue biopsy.

**Supplementary Information:**

The online version contains supplementary material available at 10.1186/s40644-024-00680-0.

## Introduction

The tumor microenvironment (TME) refers to the internal and external environment of tumor cells, including abundant infiltrating immune cells along with inflammatory cytokines. The TME plays an important role in the growth and metastasis of tumors. Recently, a considerable amount of evidence has demonstrated that immune status is closely associated with tumor progression and the patient's prognosis [1–7]. A previous study showed that the number of infiltrating neutrophils was correlated with infiltrating CD8+ T cells in muscle-invasive urinary bladder cancer. Also, high infiltration of neutrophils is associated with an immunosuppressive state, shorter survival time, and poor clinical outcomes [5]. In patients with high-grade glioma, a decrease of CD4+ T-cells and an increase of regulatory T cells drive resistance towards immunotherapy [2]. Moreover, most identified biomarkers reflect the immune characteristics of TME [8]. Among them, tumor-infiltrating immune cells and expression of immune-related molecules had been confirmed to be effective biomarkers reflecting the status of the TME [9, 10]. High expression levels of immune checkpoints might suggest an immunosuppressive TME resulting in tumor cell evasion of immune attack [11]. Thus, immune-related biomarkers of the TME are associated with tumor prognostic outcomes and therapeutic efficacy.

Traditionally, surgery, chemotherapy, and radiotherapy are the main clinical treatments for malignant tumors. Besides killing tumor cells directly, radiation therapy (RT) can activate the immune response of patients through multiple mechanisms, including the generation of neoantigens, activation of dendritic cells, and production of interferon. In addition, immunotherapy has advanced in recent years, especially immune checkpoint blockade (ICB) therapy, which has emerged as the main therapeutic option for cancer [12–15]. Hence, when RT enhances immune responses and turns cold tumors into hot tumors by changing the TME, ICB may improve the overall therapeutic efficacy when combined with RT [16]. Unfortunately, only a small proportion of patients benefit from the combination of ICB and RT [17–19]. One of the main reasons may be the lack of immune infiltration in TME, the so-called “cold tumor” [20].

In our previous study, the population of CD8+ T lymphocytes in TME exhibited different trends before and after 10F radiotherapy in cervical cancer (CC). High CD8+ T cells infiltration was correlated with increased IRF1 expression in the nucleus of tumor cells and better short-term outcomes, and there was a positive association between the expression of PD‐L1 and IRF1 in tumor cells. The tumor-infiltrating macrophages (defined as CD68+ cells) also displayed heterogeneous responses to concurrent chemoradiotherapy (CCRT) [21]. Thus, evaluation of the immune cell infiltration in TME and the immune-related gene expression during chemoradiotherapy may predict the therapeutic response and help select subgroups of patients that are more likely to benefit from immunotherapy.

Because tumor-associated immune responses happen in a short duration after RT, prompt tissue biopsies obtained at various times may be required to reveal the changes in the TME [22]. As the most common gynecologic tumor, CC serves as an ideal model to approach this problem, characterized by its strong immunogenicity and relative accessibility for biopsy. So, we developed an immunomarker classifier by screening 13 immune biomarkers in CC before treatment and after 10F of radiotherapy. The biomarker expression has to be determined by immunohistochemistry performed after tissue sample extraction, and the focal lesion gradually shrinks or even disappears after radiotherapy and/or chemotherapy. These limit its clinical application. Therefore, a noninvasive, timely, and efficient predictor for this immunomarker classifier is needed.

As an emerging technique, radiomics have been mainly applied in the early diagnosis, differential diagnosis [23, 24], staging [25, 26], prognosis, and treatment evaluation [27–31] of tumors, which have demonstrated improved diagnostic and predictive performance compared to conventional imaging techniques. It is found that radiomics could also effectively predict the immune status [32, 33]. Concentrating on the fields of radiomics and oncology [34–36], we believe that radiomics has the potential to predict this immunomarker classifier. To our knowledge, a radiomics signature based on immune scores for assessment of the infiltration of immune cells in TME and the expressions of immune checkpoint genes has not been well established.

In this study, we identified the biomarkers and established an immunomarker classifier by using mathematical models to evaluate the CCRT-induced alterations in CD8+ T cells and macrophages. Furthermore, we built a radiomics signature based on this immune model to assess the dynamic changes in tumor-infiltrating CD8+ T cells and macrophages in CC patients during CCRT.

## Methods

### Study design and patients

The study enrolled a cohort of 30 consecutive patients treated at the Cancer Hospital of Shantou University Medical College. All these patients matched the following inclusion criteria: (a) pathologically confirmed primary cervical squamous cell carcinoma (FIGO stages IB3, IIA2, IIB-IVA); (b) received CCRT; (c) standard contrast-enhanced pelvic CT and MRI scans performed within 14 days before CCRT; (d) availability of primary tumor biopsy during the period of CCRT. The exclusion criteria were as follows: (a) history of antitumor therapy (immunotherapy and radiotherapy that would cause overlapping of planned radiotherapy fields); (b)history of autoimmune diseases; (c) concurrent immunotherapy during radiotherapy; (d) contraindications for biopsy; (e) tumor lesions could not be distinguished by CT.

Enrolled patients received external beam radiation therapy (EBRT) and platinum-based concurrent chemotherapy followed by brachytherapy (see detailed regimens in the supplement). Before treatment, patients underwent essential examinations including tumor biopsy, blood tests and other examinations for tumor staging and pretreatment assessment. Another tumor biopsy and complete blood count were obtained instantly after 10F of radiotherapy. Cervical biopsies were obtained from the superior portion of the ectocervix using biopsy forceps (2-5 pieces, no less than 2*2 mm tissue). After the whole course of pelvic EBRT, short-term response to CCRT in patients was evaluated in accordance with the RECIST 1.0 version. Complete response (CR) was defined as the absence of cervical lesions as assessed by the bimanual examination. Partial response (PR) was defined as at least a 30% decrease in the lesion maximum diameter compared with the baseline.

Clinical and hematological data, such as age, FIGO staging, platelet count (PLT), absolute neutrophil count (ANC), absolute monocyte count (AMC), and absolute lymphocyte count (ALC), were derived from medical records. The neutrophil-lymphocyte ratio (NLR) was calculated as the ratio of ANC to ALC, platelet neutrophil ratio (PNR) as the ratio of APC to ANC, platelet lymphocyte ratio (PLR) as the ratio of APC to ALC, and lymphocyte monocyte ratio (LMR) as the ratio of ALC to AMC. Using the receiver operating characteristic (ROC) curves and the Youden Index, the optimal cutoff scores for hematological features were determined based on their association with the response to the CCRT. The size of the primary tumor and LN status were recorded in the clinical radiological report. Patients with visible regional LN > 1cm in the maximal short-axis diameter and/or clusters of ≥ 3 lymph nodes were identified as clinically LN-positive.

The FIGO staging was classified according to the revised 2018 International Federation of Gynecology and Obstetrics (FIGO) staging system for CC. Ethical approval was obtained from the clinical study ethics committee in the Cancer Hospital of Shantou University Medical College (Shantou, P.R. China), and all patients were informed consent. This study complied with the Declaration of Helsinki.

### IHC staining and construction of immunomarker classifier

Immunohistochemical staining and evaluation were processed as previously described [21]. We calculated the changes of expression of 13 immune biomarkers after 10F-RT, including infiltrating CD8+T cells, infiltrating CD68+ cells, and 11 IFN-responsive molecules (PD-L1, SERPINB9, CD47, nuclear IRF1, nuclear STAT1, HLA-A, HLA-B/C, β2M, TAP1, LMP2 and LMP7) in tumor cells. The least absolute shrinkage and selection operator (LASSO) regression model was used to determine the most useful predictive features out of all the 13 immune features, and then we generated a multi-immune markers-based classifier to predict patients’ response to CCRT [37]. To demonstrate the stability of this model, we applied internal ten-fold cross-validation [38, 39]. The LASSO logistic regression model was analyzed with the “glmnet” R package.

### CT acquisition and VOI delineation

The pretreatment contrast-enhanced computed tomography scan (Philips Brilliance CT Big Bore Oncology Configuration, Cleveland, OH, USA; voxel size: 1.0 × 1.0 × 5.0 mm; scan voltage: 120 kV; convolution kernel: Philips Healthcare’s B) was conducted for each patient. Patients received a cubital vein injection of iodinated contrast agent (IODAMEPOLE, 70-80ml, 1.8-2.0ml/s, 22-25s) prior to scanning. Portal venous-phase images were transmitted to Varian Eclipse TPS (Treatment Planning System), and then the volume of interest (VOI) was segmented by a senior radiation oncologist with 15 years of experience. In our study, the VOI covered the entire gross volume of the primary tumor and the whole uterus.

### Feature extraction, selection, and radiomics signature construction

From the VOI of each patient, a total of 97 image features were extracted using in-house software implemented in MATLAB (version R2016a, MathWorks, Natick, USA). The radiomics features could be separated into 3 types: 24 intensity CT features, 20 geometric, and 53 textural features (detail in supplementary). We found that the ranges of different features were varied, and hence the imaging data were standardized with zero-mean normalization (Z-score) to avoid the potential effect on the classification performance. Firstly, univariate regression analysis was used to evaluate the predictive performance of each radiomics feature, and only the features with *p* < 0.2 were selected for intensive study. Secondly, we used the LASSO model with 10-fold cross-validation to further select the features with non-zero coefficients and construct a radiomics signature to predict the previous immunomarker classifier.

### Statistical analysis

Continuous variables were compared using the Student’s t-test (normally distributed) or Wilcoxon rank-sum test (nonnormally distributed). The Chi-square test was utilized to compare the differences in categorical variables. All clinical variables were evaluated with univariate logistic regression analyses, and multivariate logistic regression was performed to determine the significant independent factors of treatment benefit. The discrimination of the immunomarker classifier and radiomics signature was quantified with the receiver operator characteristic (ROC) curve. Statistical analysis was conducted using R software (version 4.0.5), jamovi software (version 1.6.23), and SPSS software (version 23.0). A two-sided *p*-value < 0.05 was regarded as statistically significant.

## Results

### Clinical characteristics

The detailed clinical characteristics of the cohort were summarized in Table 1. All enrolled patients were well tolerated, and no patient presented with a diagnosis of malnutrition or urinary tract infection throughout the duration of the trial. Of the 30 patients included in the study, 17 (56.7%) were evaluated as CR after CCRT, and the rest of 13 (43.3%) were assessed as PR. We used the receiver operating characteristic (ROC) curves and the Youden Index to select the optimal cutoff points for all 8 hematological features (Supplementary Fig. S1, S2) and classified them into low and high groups. Between the CR and PR groups, there were statistically significant differences in FIGO stage, tumor size, pre-NLR, pre-PLR, pre-LMR, 10F-PNR, 10F-PLR, and 10F-LMR (*p* < 0.05). However, age, LN metastasis, pre-PNR and 10F-NLR showed no significant differences (Table 1).

**Table 1:** Characteristics of patients according to the treatment response

Variables	Response		p
	PR (*n* = 13)	CR (*n* =17)	
**Age (years)**			0.177
<60	10 (76.9%)	9 (52.9%)	
≥60	3 (23.1%)	8 (47.1%)	
**FIGO stage**			0.030 *
I~II	4 (30.8%)	12 (70.6%)	
III~IV	9 (69.2%)	5 (29.4%)	
**Tumor size (cm)**			0.020 *
<4	1 (7.7%)	8 (47.1%)	
≥4	12 (92.3%)	9 (52.9%)	
**LNs metastasis**			0.153
Negative	5 (38.5%)	11 (64.7%)	
Positive	8 (61.5%)	6 (35.3%)	
**Pre-NLR**			0.009 *
Low	1 (7.7%)	9 (52.9%)	
High	12 (92.3%)	8 (47.1%)	
**Pre-PNR**			0.225
Low	9 (69.2%)	8 (47.1%)	
High	4 (30.8%)	9 (52.9%)	
**Pre-PLR**			0.004 *
Low	4 (30.8%)	14 (82.4%)	
High	9 (69.2%)	3 (17.6%)	
**Pre-LMR**			0.003 *
Low	7 (53.8%)	1 (5.9%)	
High	6 (46.2%)	16 (94.1%)	
**10F-NLR**			0.127
Low	4 (30.8%)	10 (58.8%)	
High	9 (69.2%)	7 (41.2%)	
**10F-PNR**			0.012 *
Low	4 (30.8%)	13 (76.5%)	
High	9 (69.2%)	4 (23.5%)	
**10F-PLR**			0.013 *
Low	6 (46.2%)	15 (88.2%)	
High	7 (53.8%)	2 (11.8%)	
**10F-LMR**			0.004 *
Low	10 (76.9%)	4 (23.5%)	
High	3 (23.1%)	13 (76.5%)	
**Immune score**, median (interquartile range)	0.042 (-0.264 to 0.278)	0.548 (0.416 to 0.749)	0.002 *

Tumor size and LNs metastasis were evaluated by a radiologist according to patient’s MRI before treatment

*Abbreviations*: *LNs metastasis* Lymph nodes metastasis, *Pre-NLR* Neutrophil lymphocyte ratio before treatment, *Pre-PNR* Platelet neutrophil ratio before treatment, *Pre-PLR* Platelet lymphocyte ratio before treatment, *Pre-LMR* Lymphocyte monocyte ratio before treatment, *10F-NLR* Neutrophil lymphocyte ratio after 10F RT, *10F-PNR* Platelet neutrophil ratio after 10F RT, *10F-PLR* Platelet lymphocyte ratio after 10F RT, *10F-LMR* Lymphocyte monocyte ratio after 10F RT

Significance: * *p* value < 0.05

### Development and validation of immunomarker classifier

**Fig. 1:**
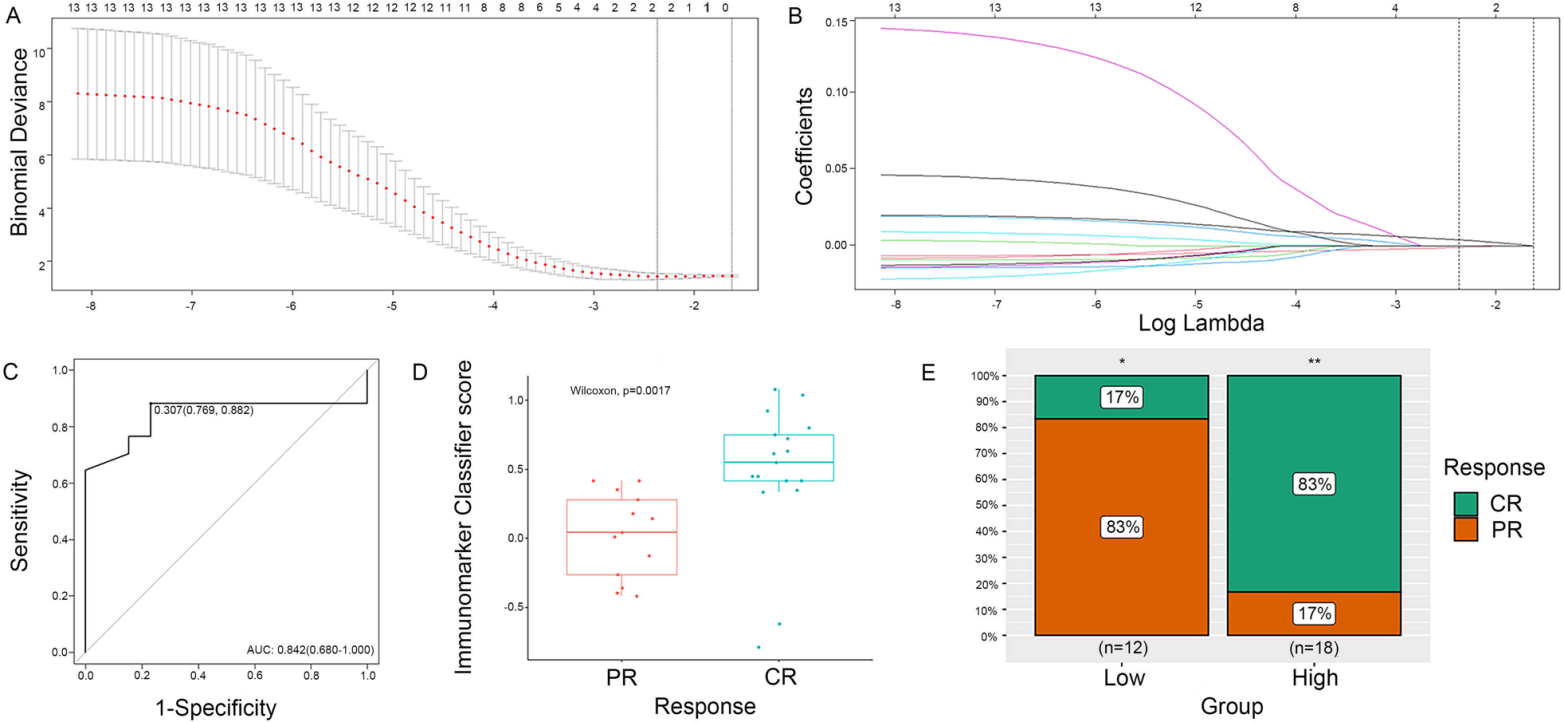
Feature selection and construction of the immunomarker classifier using LASSO logistic regression, and the predictive performance of the classifier. **A** Tuning parameter (λ) selection in the LASSO model via ten-fold cross-validation based on minimum criteria. The binomial deviance was plotted versus log (λ). The dotted vertical line was set with minimum criteria, and the optimal λ value of 0.094 with log (λ) of -2.368 was selected. **B** LASSO coefficient profiles of the 13 immune markers. A vertical line was drawn at the value selected by 10-fold cross-validation, where optimal λ resulted in 2 features with nonzero coefficients. **C** ROC curve and Youden’s index of the Immunomarker Classifier. **D** The immune score boxplots between complete and partial remission groups. **E** Comparison of the immediate responses between the low-score group and the high-score group. Significance: * *p* value < 0.05. **p < 0.01

The univariate regression analysis of immune features was listed in Supplementary Table S1, which indicated that the infiltrating CD8+T cells were the most significant variable in predicting response to CCRT in CC patients. The changes in the number of infiltrating CD8+T cells and CD68+ cells were selected to build the final immunomarker-based classifier by using the LASSO logistic regression model with ten-fold cross-validation (Fig. 1A, 1B). The classifier showed a satisfying discriminatory efficacy for treatment response with AUCs of 0.842 (95% CI, 0.680-1.000) (Fig. 1C). The model score for each patient was calculated according to the following formula: immune score = 0.4569 + 0.0041 × (CD8+T cell at 10F minus that at baseline) - 0.0009 × (CD68+ cell at 10F minus that at baseline), and the immune scores (median [interquartile range]) in patients with CR after CCRT were significantly higher than those with PR (0.548 [0.416 to 0.749] vs. 0.042 [-0.264 to 0.278], respectively, *p* = 0.0017) (Table 1 and Fig. 1D). As displayed in Fig. 1C, the optimal cut-off for classifier scores determined by Youden’s index was 0.307, and the model had a sensitivity of 0.882 while obtaining a specificity of 0.769, respectively. Accordingly, patients could be divided into two groups: low-score group (score < 0.307) and high-score group (score ≥ 0.307). The low-score group consisted of 12 patients, in which 2/12 (17%) were evaluated as CR and 10/12 (83%) as PR. For the high-score group, 15/18 (83%) patients were considered as CR, and the remaining 3/18 (17%) as PR. High-score patients had a significantly higher CR rate than low-score patients (*p* < 0.001) (Fig. 1E).

We performed univariate binary logistic regression analysis to evaluate the ability of clinical features to distinguish therapy response. As shown in Table 2, the AUC values of this immunomarker-based model were higher than any single clinical variable, which suggested that this classifier achieved the best predictive efficacy. Multivariate logistic regression was performed adjusting for clinical variables and it suggested that the immunomarker classifier score was an independent predictor of treatment response (Table 2).

**Table 2:** Risk factors for treatment response in cervical cancer

Variable	Univariate Logistic Regression			Multivariate Logistic Regression	
	OR (95% CI)	p	AUC	OR (95% CI)	p
**Immune score** (per 0.1 increase)	12.267 (1.493-100.770)	0.020 *	0.842	32.448 (1.430-735.592)	0.029 *
**Age (years)** (<60 vs. ≥60)	2.963 (0.596-14.730)	0.184	0.620	NA	NA
**FIGO stage** (I~II vs. III~IV)	0.185 (0.038-0.893)	0.036 *	0.699	NA	NA
**Tumor size (cm)** (<4 vs. ≥4)	0.094 (0.010-0.891)	0.039 *	0.697	NA	NA
**LNs metastasis** (Negative vs. Positive)	0.341 (0.076-1.522)	0.159	0.631	NA	NA
**Pre-NLR** (Low vs. High)	0.074 (0.008-0.704)	0.023 *	0.726	NA	NA
**Pre-PNR** (Low vs. High)	2.531 (0.557-11.512)	0.229	0.611	NA	NA
**Pre-PLR** (Low vs. High)	0.095 (0.017-0.529)	0.007 *	0.758	NA	NA
**Pre-LMR** (Low vs. High)	18.667 (1.879-185.406)	0.012 *	0.740	36.366 (1.050-1255.634)	0.047 *
**10F-NLR** (Low vs. High)	0.311 (0.068-1.430)	0.133	0.640	NA	NA
**10F-PNR** (Low vs. High)	0.137 (0.027-0.695)	0.016 *	0.729	NA	NA
**10F-PLR** (Low vs. High)	0.114 (0.018-0.716)	0.020 *	0.710	NA	NA
**10F-LMR** (Low vs. High)	10.833 (1.961-59.836)	0.006 *	0.767	20.348 (1.100-377.655)	0.043 *

*Abbreviations*: *NA* Not available, *LNs metastasis* Lymph nodes metastasis, *Pre-NLR* Neutrophil lymphocyte ratio before treatment, *Pre-PNR* Platelet neutrophil ratio before treatment, *Pre-PLR* Platelet lymphocyte ratio before treatment, *Pre-LMR* Lymphocyte monocyte ratio before treatment, *10F-NLR* Neutrophil lymphocyte ratio after 10F RT, *10F-PNR* Platelet neutrophil ratio after 10F RT, *10F-PLR* platelet lymphocyte ratio after 10F RT, *10F-LMR* Lymphocyte monocyte ratio after 10F RT

Significance: * *p* value < 0.05

### Construction and performance of radiomics signature

**Fig. 2:**
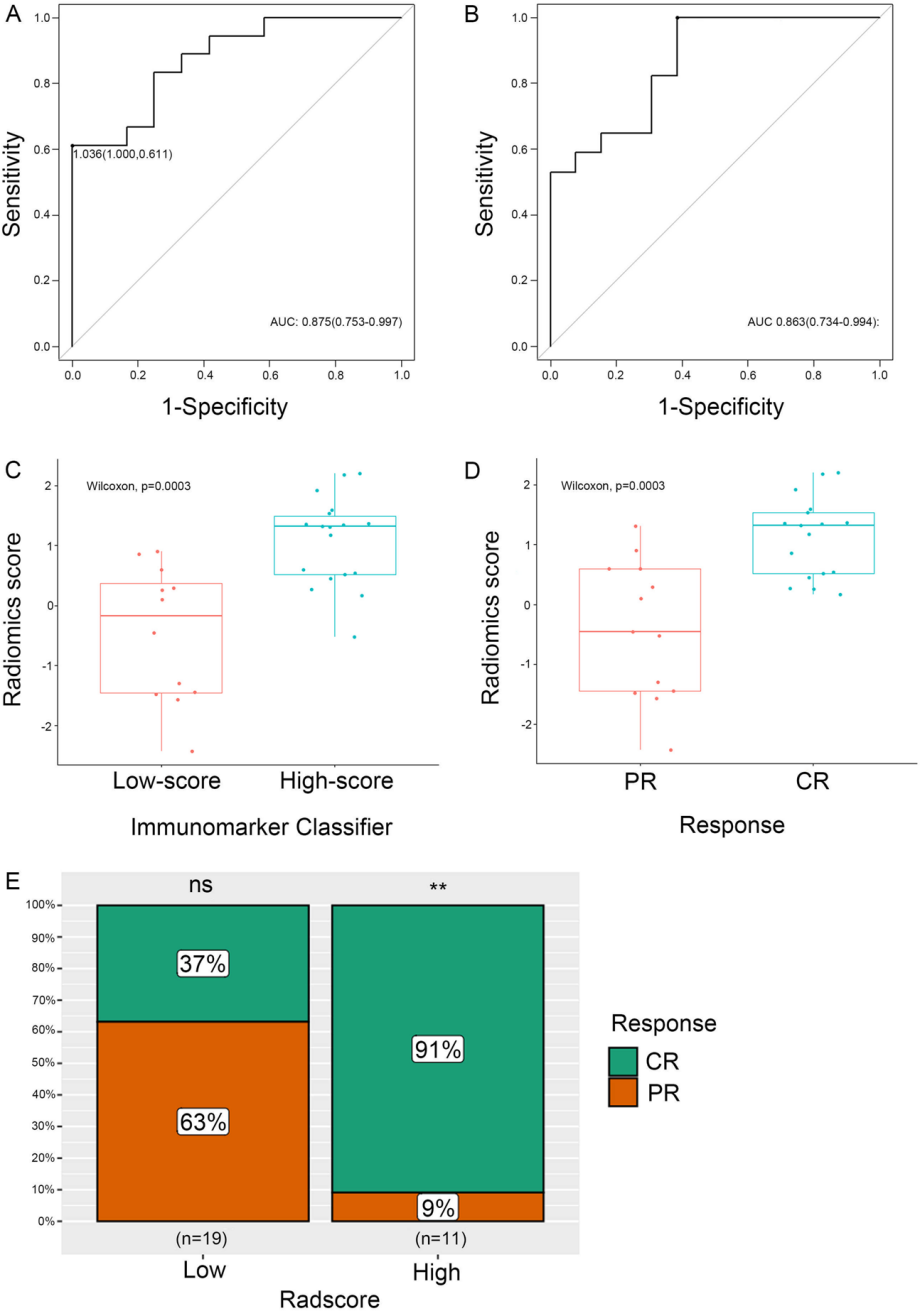
Construction and predictive performance of the radiomics signature using LASSO logistic regression. **A** ROC curves of the Radiomics Signature to predict Immunomarker Classifier. **B** ROC curves of the Radiomics Signature to predict treatment response. **C** The radiographic scores boxplots between the high-score immune group and the low-score group. **D** The radiographic scores boxplots between complete and partial remission groups. **E** Comparison of the immediate responses between the low-Radscore group and the high-Radscore group. Significance: ns = not significant, **p < 0.01

Of all 97 radiomics features extracted from VOI, there were 36 features meeting the criterion that *p* < 0.2 in univariate analysis. LASSO regression algorithm with ten-fold cross-validation, a common regression model for high-dimensional data, was utilized to further determine the most significant 4 imaging features and develop a radiomics signature to discriminate immunomarker classifier. An AUC of 0.875 (95% CI, 0.753-0.997) revealed that the radiomics signature had a great ability to distinguish the high-score group from the low-score group (Fig. 2A). Fig. 2B showed that the radiomics signature also exhibited good discrimination for therapy response with AUCs of 0.864 (95% CI, 0.734-0.994). The radiomics signature and 10F-LMR were identified as independent factors of treatment response in CC patients by multivariate regression analysis (Supplementary Table S2).

The calculated formula of the radiomics score was shown in the supplementary. There were significant differences between the radiomics score (median [interquartile range]) in immunomarker groups (-0.175 [-1.450 to 0.371] vs. 1.320 [0.519 to 1.490], respectively, *p* = 0.0003) and response groups (-0.452 [-1.450 to 0.595] vs. 1.320 [0.512 to 1.530], respectively, *p* = 0.0004) (Figs. 2C and 2D). The optimal cut-off value of radiomics score determined by ROC curve analysis was 1.036, and patients were classified into low-Radscore group (Radscore < 1.036) and high-Radscore group (Radscore ≥ 1.036). The low-Radscore group included 19 patients, in which fewer patients received CR but there is no significant difference (37% vs. 63%, *p* > 0.05). As for the high-Radscore group, most patients were evaluated as CR (91% vs. 9%, *p* < 0.01). It indicated that treatment response was significantly different for patients stratified by radiomics signature (*p* = 0.004) (Fig. 2E).

## Discussion

Tumor progression and response to therapy are associated with TME. It has been reported that chemoradiotherapy could exert anti-tumor efficiency by altering the TME [40–42]. Similarly, our previous study showed that the variations of innate immune markers during the treatment course displayed a closer relation to the efficacy of CCRT for CC, demonstrating that the alternations in the TME resulting from chemoradiotherapy may be an important factor for affecting patient prognosis and therapeutic response. In this study, we proposed an immunomarker classifier to appraise the alterations in TME-related biomarkers during therapy in CC patients, and a corresponding radiomics signature was created.

Based on the clinical research data from our previous trial (NCT03744819), we analyzed the changes of the expression of 13 immune markers before and after 10 sessions of radiotherapy, selected the most useful predictive features via the LASSO algorithm with ten-fold cross-validation, and developed a new immunomarker classifier. Patients with lower immune scores had a lower CR rate, indicating the immune score may be a poor prognostic factor. We also confirmed that the robust value of the immunomarker classifier was an independent predictor of treatment response. Furthermore, compared with the FIGO staging system, which was widely used to guide treatment strategy for CC, this immunomarker classifier could provide additional information about the infiltration of immune cells and show good predictive ability.

In this study, the immunomarker classifier was determined by the number of changes in CD8+ T cells and CD68+ cells in the TME during CCRT. CD8+ T cells are cytotoxic T lymphocytes that secrete various cytokines to exert their antitumor effect [43]. CD68 is a specific surface marker of macrophages. CD8+ T cells and tumor-associated macrophages (TAMs) are the predominant immune population in the TME. Although macrophages have several function phenotypes, the majority of macrophages tend to acquire the M2 phenotype and facilitate tumor growth and metastasis [44, 45]. A study demonstrated that the infiltrating lymphocyte percentage in breast cancer was an independent predictor of the efficacy of neoadjuvant chemotherapy [46]. Another study on lung cancer found that tumor-infiltrating lymphocytes were associated with the efficacy of immune checkpoint inhibitors [47]. Besides, among patients with advanced colorectal cancer who had received bevacizumab plus irinotecan/oxaliplatin-based combination chemotherapy, those with lower TAM infiltration showed two times longer overall survival compared to those with higher TAM infiltration [48]. These findings suggest that infiltrating lymphocytes play a fundamental role in anti-tumor immune response and prognosis, while TAMs favor tumor progression.

The same trend was found in this study. Patients with higher scores had increased CD8+ T cells and decreased CD68+ cells after CCRT in the TME which we had termed immune-inflamed TME (i.e., “hot” tumor). And vice versa, patients with lower scores had an immunosuppressive TME (i.e., “cold” tumor). In short, CC patients could be divided into two different groups based on the immunomarker classifier score for predicting the immune infiltration to guide individualized treatment.

In the present work, we performed a tumor biopsy before and after 10F radiotherapy to evaluate the changes in the expression of the biomarkers related to the TME. This approach has the advantage of discovering the immuno-phenotype changes and the molecular changes during chemoradiotherapy. Hence, our immunomarker classifier calculated immune scores by analyzing changes in the number of CD8+ T cells and macrophages between pre- and post-treatment comprehensively.

The detection of immune-related biomarkers in TME requires biopsies which is not feasible in most disease types and is a lack of compliance in patients with accessible tumors. Radiomics is currently in development and can explore diagnostic and prognostic information by extracting tumor features quantitatively from medical images in the field of tumor research which may solve this problem [49]. Currently, several studies have investigated the relationship between radiomics signatures and TME. The above-mentioned study by Sun et al [32], and Jiang et al [50] found that radiomics signatures could reflect the immune state and the characteristics of the TME. In this study, we tried to establish the radiomics signature in CC via CT to predict the immunomarker classifier. At the same time, we found that the radiomics signature could identify CC patients who have a better treatment response, and the patients with higher radiomics scores had a significantly higher CR rate than those with lower radiomics scores.

In recent years, immunotherapy represented by immune checkpoint blockade (ICB) has been a significant advance in the treatment of various tumors. Nevertheless, only a small part of patients display improved responses from the PD-L1 blockade or even RT and ICB combined therapy. Consequently, aiding the identification of patients suitable for immunotherapy by assessing the immune status following chemoradiation therapy is necessary to avoid overtreatment and unnecessary economic burden. The result of the present study demonstrated that the high-Radscore group displayed increased CD8 + T cells infiltration and decreased macrophages infiltration in the TME after 10F-RT, meaning that patients were in an immunocompetent state, while the low-Radscore group had the opposite result. We envisaged that the dynamic modifications of the tumor-infiltrating immune cells in patients may be predicted by performing contrast-enhanced pelvic CT during chemoradiotherapy. It is a potential tool in guiding individualized treatment regimens for cancer patients.

This study had several limitations. Firstly, this was a single-center study and the data set was not divided into training and testing cohorts due to the limited sample size. Ten-fold cross-validation was used to internally verify to reduce model errors and prevent overfitting. Thus, further large clinical research is warranted. Secondly, it has been reported that M1 and M2 macrophages co-exist in TME [51]. CD68 cannot distinguish between two phenotypes but is rather a pan-macrophage marker. Further experiments are needed to discriminate the impact of two phenotypes, such as flow cytometry and single-cell sequencing.

## Conclusions

In conclusion, the immunomarker classifier could assess CD8+ T cells and macrophages infiltration in TME during CCRT in CC. The radiomics signature could predict the immune score to evaluate the dynamic changes in the infiltration of CD8+ T cells and macrophages during CCRT. Additionally, the radiomics signature might be a potential predictive tool to guide the stratification of treatment strategies and achieve treatment individualization for patients.

### Supplementary Information

**Additional file 1:** **Supplementary Methods and Results**. **Table S1.** Univariate logistic regression analysis for immune features. **Table S2.** Independent predictors for treatment response in cervical cancer. **Fig.** **S1.** ROCcurves and Youden Index of hematological features before treatment. **Fig. S2.** ROC curves and Youden Index of hematological features after 10F RT.

## Data Availability

The datasets used and/or analysed during the current study are available from the corresponding author on reasonable request.

## Abbreviations

AUC: Area Under Curve
CCRT: Concurrent chemoradiotherapy
CT: Computed Tomography
EBRT: External beam radiation therapy
FIGO: International Federation of Gynecology and Obstetrics
ICB: Immune checkpoint blockade
LASSO: Least Absolute Shrinkage and Selection Operator
MRI: Magnetic Resonance Imaging
RECIST: Response Evaluation Criteria in Solid Tumors
ROC: Receiver operating characteristic curve
TAM: Tumor-associated macrophage
VOI: Volume of interest
