# Supplementary material for: Radiomics signature for dynamic changes of tumor-infiltrating CD8+ T cells and macrophages in cervical cancer during chemoradiotherapy
Source: Cancer Imaging. 2024 Apr 23;24:54. doi: 10.1186/s40644-024-00680-0 (PMC11036574; doi:10.1186/s40644-024-00680-0)
Supplement: Supplementary file 1 — Additional file 1: Supplementary Methods and Results. Table S1. Univariate logistic regression analysis for immune features. Table S2. Independent predictors for treatment response in cervical cancer. Fig. S1. ROCcurves and Youden Index of hematological features before treatment. Fig. S2. ROC curves and Youden Index of hematological features after 10F RT. [file 40644_2024_680_MOESM1_ESM.docx]

**Supplementary Information**

**Supplementary Methods and Results**

**Treatment**

Enrolled patients were subjected to concurrent chemoradiotherapy (CCRT). Radiotherapy consisted of external beam radiation therapy (EBRT) (median dose 46Gy/23 fractions (F)) to the whole pelvis followed by EBRT boost (median dose 14Gy/7F) to positive lymph nodes and brachytherapy boost (24-32Gy/4-5 fractions) to the primary tumor and surrounding subclinical disease. During EBRT, patients also received platinum-based concurrent chemotherapy. The chemotherapy regimen consisted of either weekly cisplatin [30-40 mg/m^2 (body surface area)] or a combination of cisplatin (25 mg/m^2, day 1-3) and fluorouracil (0.5 g/m^2, day 1-4) **every three weeks for 2 cycles**.

**Radiomics features**

The intensity features were obtained from the histogram of the voxel intensities of the delineated structures e.g., mean represents the average voxel intensity and the skewness quantifies the degree of asymmetry around the mean value. The geometric features, such as volume, bounding-box-volume and major-axis-length, were extracted from the three-dimensional (3D) contoured structures. The textural features were defined to quantify the heterogeneity of tissue and they were derived from three different matrices: the gray level co-occurrence matrix (GLCM), gray level run-length matrix (GLRLM), neighboring grey-tone difference matrix (NGTDM) and gray level size-zone matrix (GLSZM).

**Calculated formula of radiomics score**

Radiomics score = 0.455 - (0.350 × max) + (0.437 × cshad_GLCM_mean) – (0.564 × busyness_NBH) – (0.812 × gray_level_nonuniformity_SZ)

**Supplementary Tables**

**TABLE S1.** Univariate logistic regression analysis for immune features.

| Immune features | Univariate Logistic Regression | | |
| --- | --- | --- | --- |
|  | OR (95% CI) | p | AUC |
| CD8+ cell | 1.008 (1.000-1.017) | 0.044* | 0.760 |
| CD68+ cell | 0.997 (0.993-1.001) | 0.164 | 0.570 |
| PD-L1 | 1.006 (0.980-1.032) | 0.665 | 0.590 |
| SERPINB9 | 1.001 (0.988-1.015) | 0.842 | 0.534 |
| Nuclear STAT1 | 1.011 (0.988-1.035) | 0.341 | 0.568 |
| Nuclear IRF1 | 1.013 (0.991-1.035) | 0.253 | 0.658 |
| HLA-A | 1.007 (0.982-1.034) | 0.575 | 0.511 |
| HLA-B/C | 1.002 (0.975-1.030) | 0.897 | 0.425 |
| β2M | 1.003 (0.980-1.027) | 0.781 | 0.457 |
| TAP1 | 0.994 (0.960-1.029) | 0.715 | 0.548 |
| LMP2 | 1.008 (0.967-1.051) | 0.718 | 0.545 |
| LMP7 | 1.086 (0.930-1.268) | 0.297 | 0.579 |
| CD47 | 1.013 (0.976-1.050) | 0.499 | 0.633 |

Significance: * p value < 0.05.

**TABLE S2.** Independent predictors for treatment response in cervical cancer.

| Variable | Multivariate Logistic Regression | |
| --- | --- | --- |
|  | OR (95% CI) | p |
| **Radiomics score**  (per 0.1 increase) | 13.757 (1.498-126.314) | 0.020* |
| **10F-LMR**  (Low vs. High) | 34.196 (1.822-641.933) | 0.018* |

Abbreviations: 10F-LMR: lymphocyte monocyte ratio after 10F RT.

Significance: * p value < 0.05.

**Supplementary Figures**


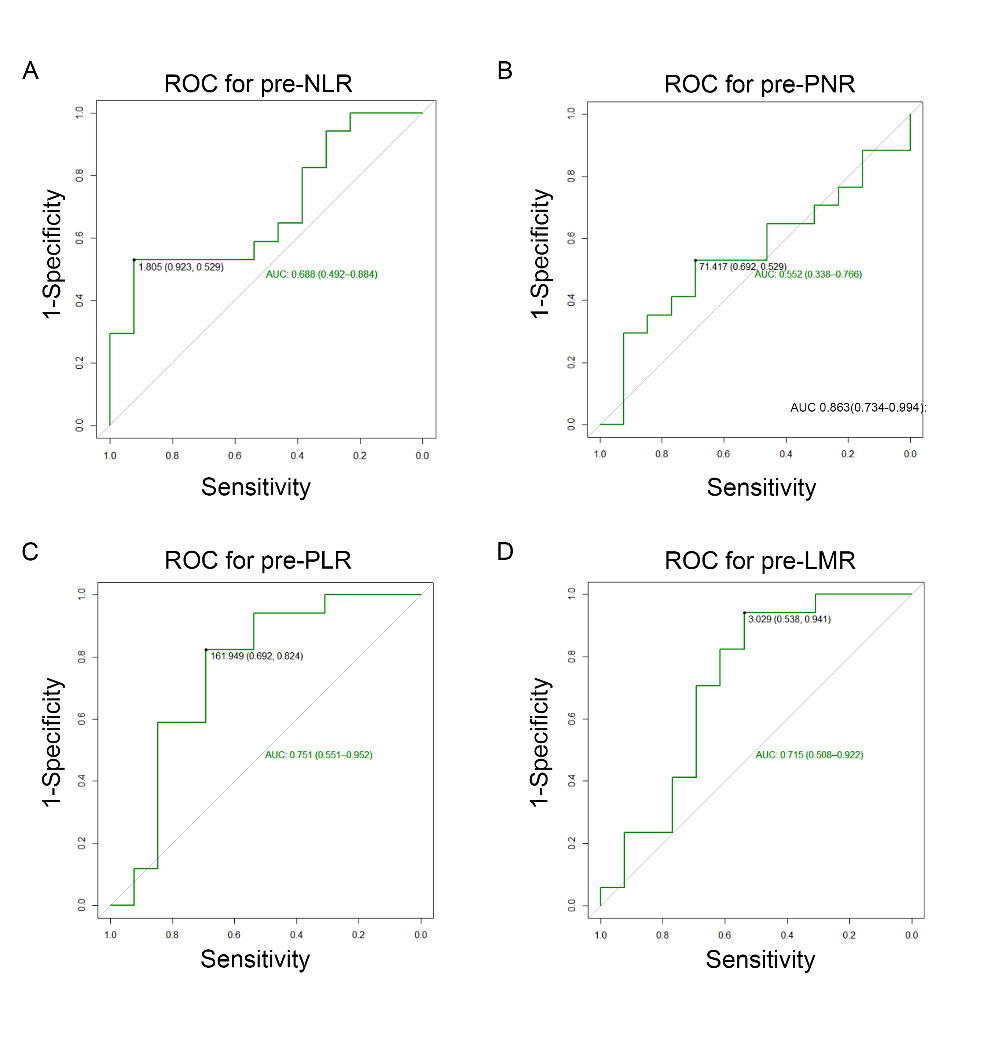


**FIG. S1** ROC curves and Youden Index of hematological features before treatment.

Abbreviations: Pre-NLR: neutrophil lymphocyte ratio before treatment; Pre-PNR: platelet neutrophil ratio before treatment; Pre-PLR: platelet lymphocyte ratio before treatment; Pre-LMR: lymphocyte monocyte ratio before treatment.


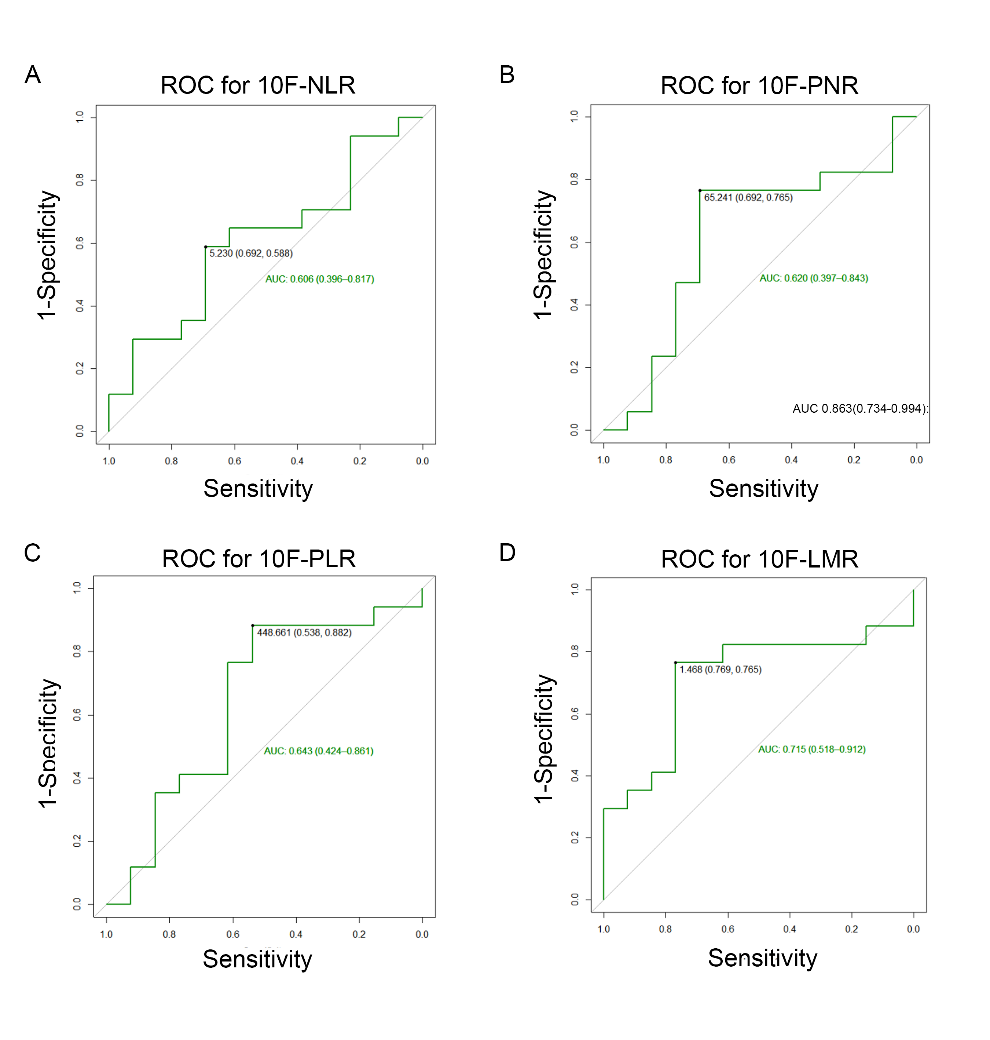


**FIG. S2** ROC curves and Youden Index of hematological features after 10F RT.

Abbreviations: 10F-NLR: neutrophil lymphocyte ratio after 10F RT; 10F-PNR: platelet neutrophil ratio after 10F RT; 10F-PLR: platelet lymphocyte ratio after 10F RT; 10F-LMR: lymphocyte monocyte ratio after 10F RT.
